# Electroencephalography Signal Processing: A Comprehensive Review and Analysis of Methods and Techniques

**DOI:** 10.3390/s23146434

**Published:** 2023-07-16

**Authors:** Ahmad Chaddad, Yihang Wu, Reem Kateb, Ahmed Bouridane

**Affiliations:** 1 School of Artificial Intelligence, Guilin University of Electronic Technology, Guilin 541004, China; 2 The Laboratory for Imagery, Vision and Artificial Intelligence, Ecole de Technologie Supérieure, Montreal, QC H3C 1K3, Canada; 3 College of Computer Science and Engineering, Taibah University, Madinah 41477, Saudi Arabia; 4 Centre for Data Analytics and Cybersecurity, University of Sharjah, Sharjah 27272, United Arab Emirates

**Keywords:** EEG, signal processing, machine learning

## Abstract

The electroencephalography (EEG) signal is a noninvasive and complex signal that has numerous applications in biomedical fields, including sleep and the brain–computer interface. Given its complexity, researchers have proposed several advanced preprocessing and feature extraction methods to analyze EEG signals. In this study, we analyze a comprehensive review of numerous articles related to EEG signal processing. We searched the major scientific and engineering databases and summarized the results of our findings. Our survey encompassed the entire process of EEG signal processing, from acquisition and pretreatment (denoising) to feature extraction, classification, and application. We present a detailed discussion and comparison of various methods and techniques used for EEG signal processing. Additionally, we identify the current limitations of these techniques and analyze their future development trends. We conclude by offering some suggestions for future research in the field of EEG signal processing.

## 1. Introduction

Brain science has become an essential field of study to unravel the mysteries of life due to developments in biomedical technology and our growing understanding of the brain [1]. Since the middle of the 20th century, the complexity of the brain has been studied and, in recent years, brain science has continued to be a hot topic for research [2]. The analysis of brain electrical activity is one of the main areas of interest in brain science [3]. As a result, the electroencephalogram (EEG) is important to analyze brain science and is often used in various brain-related research domains [4,5].

EEG is a noninvasive neuroimaging technique that involves the placement of electrodes on the scalp to record electrical activity of the brain [6]. This enables researchers to measure and analyze the electrical signals generated by the brain. These signals offer valuable information on the operating mechanisms of the brain, covering the identification of various neurological disorders and the exploration of cognitive processes such as perception, attention, and memory. EEG has gained widespread popularity as a means of investigating electrical activity of the human brain, due to its noninvasive and safe characteristics [7]. In addition, EEG signals have the potential to be integrated with other imaging modalities, including magnetic resonance imaging (MRI) [8,9], functional near-infrared spectroscopy (fNIRS) [10,11,12], and positron emission tomography (PET) [13], in order to achieve a better understanding of brain function and structure.

EEG is a signal pattern that is obtained by amplifying and recording the spontaneous biological potential of the brain on the scalp. This potential has been shown to reflect the macroscopic activity of the brain surface and is typically acquired using noninvasive electrodes applied onto the scalp. These electrodes capture the inherent and periodic electrical impulses generated by clusters of brain cells [14]. Currently, EEG is widely used in the field of neuroscience and has the potential to advance brain–computer interfaces, facilitate emotion detection, and help in partial paralysis rehabilitation [15,16]. Furthermore, EEG is a valuable tool for clinicians and researchers in identifying brain dysfunction-associated diseases, including but not limited to Alzheimer’s disease [17,18], epilepsy, schizophrenia, Creutzfeldt–Jakob disease [19], cerebral palsy [20], and cognitive impairment [21].

**Figure 1:**
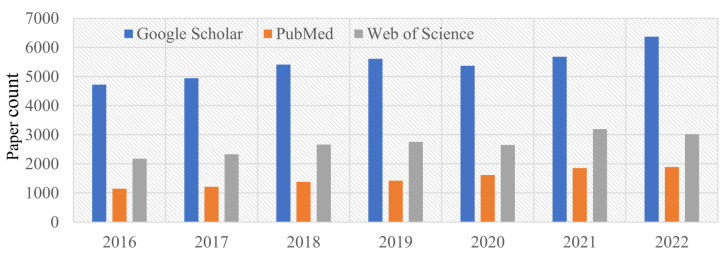
Bar graph illustrating the number of published papers over the years obtained from Google Scholar, PubMed, and Web of Science searches for the subject of the electroencephalogram (EEG). The search query used was “EEG” in the title.

Accurately identifying and analyzing EEG signals requires a thorough understanding of their complex and theoretical properties, as well as the extraction of relevant features for the given task. However, EEG signals pose significant challenges due to their unique characteristics. According to [22], one such challenge is their susceptibility to noise interference, resulting in a low signal-to-noise ratio. Additionally, their nonlinearity and lack of conformity to a normal distribution distinguish them from conventional signals. Moreover, individual factors such as age, psychology, and testing environment can cause significant variations in EEG signals [23]. In [22], it is observed that the unique properties of EEG signals pose a challenge in extracting pertinent information about specific tasks directly from them. Therefore, it is imperative to develop various methodologies for signal analysis and investigate machine learning techniques for signal analysis to better understand EEG signals [24]. Accurate extraction of relevant information on specific tasks from EEG signals requires careful consideration of their distinctive characteristics and the advancement of sophisticated signal analysis methodologies. As emphasized in [25], precise detection and analysis of EEG signals are crucial to advance our understanding of brain functioning. The high interest in EEG as a research domain is apparent in the Google Scholar, PubMed, and Web of Science search results obtained between 2016 and 2022, as illustrated in Figure 1. Our paper presents a novel contribution through a comprehensive description of denoising techniques, which includes mathematical formulations with pseudocodes. In addition, we report the recent advancements in the field of EEG, while highlighting current challenges and discussing future trends.

The primary contributions of this paper can be summarized as follows.

- We present a detailed examination of the EEG signal analysis process, including the stages of signal acquisition, denoising, and feature engineering.
- The procedure used to denoise the EEG signal is described in full, along with the accompanying evaluation standards.
- We examine feature engineering in detail in this paper, looking at time–frequency, high-order spectral, and nonlinear dynamic analysis.
- We give a thorough analysis of both traditional and deep learning methods for categorizing EEG signals. We also provide an overview of the typical datasets utilized for EEG signal processing.
- We highlight current issues with EEG signal processing techniques and offer potential solutions as well as future research prospects.

The structure of this paper is as follows. Section 2 presents a brief description of the impact of EEG acquisition as a noninvasive biomedical device. It gives a full evaluation of various denoising techniques, and an explanation of the merits and downsides of each. Additionally, it examines the significance of feature engineering and its various techniques, as well as the use of machine learning- and deep learning-based classifiers. In Section 3, future perspective and limitations are discussed. The article ends in Section 4, where the research efforts and contributions of this investigation are outlined.

## 2. The Pipeline of EEG Signal Analysis

**Figure 2:**
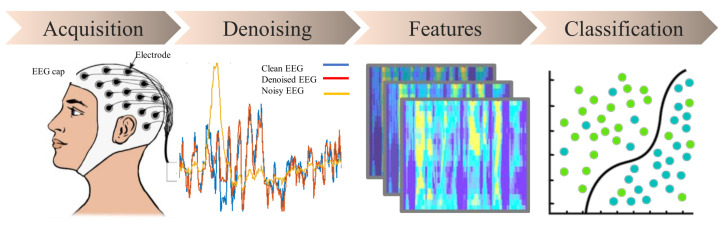
The four-step process for EEG signal analysis. The EEG signal analysis involves four stages: acquisition, denoising, feature engineering, and classification.

In this section, the focus is on the methodology of EEG signal analysis, as illustrated in Figure 2. The pipeline is related to the classification of EEG signals. The discussion begins with an explanation of the methodology of EEG signal acquisition using equipment. Then, the algorithm for denoising EEG signals is examined, which is capable of eliminating erroneous data and extracting relevant information. Next, the feature engineering process is discussed, which involves removing the less important features. Finally, the use of deep learning and machine learning algorithms for classification is explored.

### 2.1. Acquisition

EEG is a neurophysiological technique used to measure and quantify neural activity in various regions of the brain. The brain consists of a large number of neurons and their activities generate distinct scalp potentials, producing signals in different states of alertness, response to external stimuli, and other factors unique to each individual [26]. To obtain data sources for various applications and research on EEG signals, appropriate acquisition equipment is essential. Acquisition of EEG signals can be classified into two primary categories: invasive and noninvasive [27]. Invasive acquisition involves surgical insertion of electrodes into the cerebral cortex or other regions of the brain to capture signals. On the other hand, noninvasive techniques use EEG sensors positioned on the scalp’s surface and do not require implanted electrodes. Currently, most EEG signal acquisition methods are noninvasive. In Algorithm 1, we provide the main steps of EEG signal analysis.

During the initial stages of the acquisition of EEG signals, the German scientist Hans Berger detected electrical signals in the cerebral cortex via a galvanometer in 1924. After preliminary investigations, the scientists began implanting metallic electrodes directly into the cerebral cortex to capture comprehensive EEG readings. With advancements in computer technology, EEG signal collection techniques have improved significantly, leading to a higher resolution of collected EEG signals. Most EEG signal acquisition instruments that have reached relative maturity currently employ PC displays, wired data transmission, and external power sources. These devices exhibit robust data processing capabilities, favorable outcomes, and consistent performance. However, they have a heavy form factor, pose potential hazards, and exhibit high power consumption. Consequently, the need for portable EEG collection equipment has been observed [28].
**Algorithm** **1** Pipeline of EEG signal analysis**Input:** EEG signal eeg(t). **procedure** Acquisition (eeg(t)):
     Sampling equipment selection.     Choose invasive or noninvasive acquisition.     Choose the best reference electrode.     **Return**: EEG data eeg(t). **procedure** Denoising (eeg(t)):
     Using different denoising methods to process EEG signals.     **Return**: Denoised EEG signal eeg${\left(t\right)}_{new}$. **procedure** Feature_Engineering (eeg(t)):
     Perform time–frequency, high-order spectrum analysis or nonlinear analysis.     **Return**: More expressive features, eeg(t). **procedure** Classification (eeg(t), choice):
     Perform classification tasks based on choice:     **If** choice equal traditional approach **do**
          Using KNN, SVM, ... to classify.     **If** choose the same deep learning approach **do**
          Using CNN, GAN, ... to classify.     **Return**: Accuracy, precision, etc.


Portable EEG acquisition devices such as the Emotiv EPOC have gained popularity in recent years. The Emotiv EPOC utilizes nonimplantable electrodes and comprises 14 electrical data acquisition channels and two reference electrodes. Stytsenko et al. [29] found that the Emotiv EPOC can collect real EEG data. Emotiv EPOC neural headphones are also available on the market to measure brain activity [30]. However, the performance of the Emotiv EPOC headset devices, while capable of recording EEG data, is inferior to that of larger devices [31]. In another study, Martins proposed a wearable EEG acquisition device and a sleep inertia detection system of the data analysis platform [32]. The system is a monolithic low-power with a low-noise analog front-end EEG acquisition system [33]. The system has demonstrated high precision and high reliability, and flexible adjustment. Moreover, a new waterproof, lightweight, and portable EEG acquisition device was proposed to acquire and analyze the EEG signals of dolphins [34]. The device was designed to enable relatively unrestricted EEG acquisition. Their acquisition device is equipped with customized suction cups with embedded electrodes. It also incorporates a Bluetooth module to communicate with the ground station. Furthermore, they used the portable Muse brain wave sensor device for stroke identification [35]. The device follows the international 10–20 system and utilizes four recording electrodes (AF7, AF8, TP9, TP10) and one reference electrode (Fpz). In [36], they designed a high precision portable EEG acquisition system using the CompactPCI platform to address the limitations of existing EEG acquisition systems, such as high costs and limited accuracy. Aside from the previous methods, there are many ways to collect EEG signals. For example, deep brain stimulation was performed through the use of neural electrodes that were placed in specific target regions of the brain [37]. These electrodes generate current or voltage through an implantable pulse generator. Furthermore, the MR signal has the ability to reflect both oxygen saturation and blood flow in the brain [38]. It can reflect the activity of neurons and serve the purpose of functional imaging.

**Figure 3:**
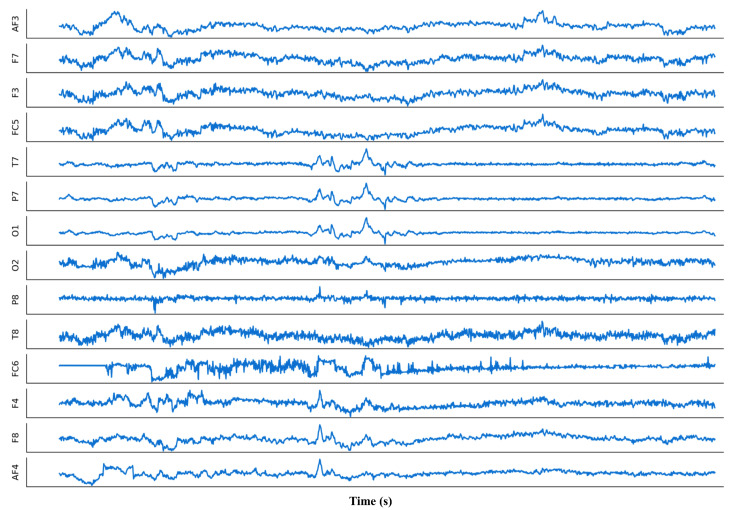
An example of 14 EEG signal channels, where the x-axis denotes time and the y-axis represents the magnitude of the 14 signals [39]. These signals can be characterized by their frequency, which refers to the number of cycles per second (Hz) of the electrical activity. These channels are named AF3, F7, F3, FC5, T7, P7, O1, O2, P8, T8, FC6, F4, F8, and AF4, which correspond to the specific electrode placements on the Emotiv Epoc equipment.

Figure 3 shows a short segment of an EEG signal [39] recorded using a 14-channel Emotiv Epoc device. These 14 EEG signals are specific locations on the scalp where electrodes are placed to measure brainwave activity. In general, the frequency of an EEG signal is measured in Hz and refers to the number of cycles that occur per unit time [40]. Specifically, there are five waves that correspond to five ranges of frequencies in EEG signals [41], namely, Delta [0.5 to 4 Hz], Theta [4–8 Hz], Alpha [8–13 Hz], Beta [13–30 Hz], and Gamma [>30 Hz]. Delta waves are related to slow-wave activity in the brain and deep sleep; Theta waves are commonly observed during periods of relaxation and meditation; Alpha waves are most noticeable when the eyes are closed; Beta waves are associated with cognitive processing that is actively engaged and can be detected during tasks that require significant attention; and Gamma waves are linked to advanced cognitive processing and the merging of sensory information. In addition, each EEG channel is named according to its location relative to the midline of the head and its distance from the forehead or back of the head. Together, these channels allow researchers and clinicians to measure electrical activity in various regions of the brain, and gain insight into cognitive processes such as attention, memory, and emotion.

Table 1 presents a summary of recent studies on EEG signal analysis in different application domains. In [42], commercial EEG equipment was used to collect signals to analyze driver fatigue. A third-order Butterworth bandpass filter was used to remove irrelevant information from the raw signals [42]. Another study by Gamage et al. utilized the Emotiv EPOC to detect the driver’s emotions while driving and extracted features using EEGlab and other tools in Matlab. The data was then classified to develop an emotion classification model aimed at reducing the risk of car accidents. The study demonstrated the high reliability of the dataset collected by the device [43]. Table 1 provides relevant details, such as the study objective, data source, and data processing techniques used in the different studies.

### 2.2. Denoising

As mentioned above regarding the acquisition of EEG signals, multiple electrodes are placed on the scalp. However, external interference can cause diverse artifacts to emerge, which can compromise the quality of the signals. Physiological artifacts, such as involuntary eye movements, blinking, heart activity, and muscle movement, are known to be present in EEG signals and can negatively affect their quality [54]. Therefore, denoising EEG signals has become a topic of significant research interest and attention. To ensure the reliability of features extracted from EEG signals, it is essential to remove any associated artifacts. Currently, several denoising techniques have been developed.

#### 2.2.1. Regression Method

The traditional approach to remove eye artifacts in EEG signals is the regression-based analysis approach. During the recording of the EEG signal, an electro-oculogram is recorded concurrently to obtain coefficients for various noise sources such as blinking artifacts (VEOG), eye movement artifacts (HEOG), and other noise sources, by using regression analysis. These coefficients estimate the ratio of artifacts in a particular EEG channel [55]. In [56], they proposed a general lagged regression model to represent this process as follows.

(1) $$\mathrm{eeg}\left(t\right)=EEG\left(t\right)-\sum_{g=0}^{T}\beta_{g}\mathrm{eog}\left(t-g\right)$$

where eeg(*t*) and eog(*t*–*g*) are the recorded EEG and EOG information at times *t* and t˘g, respectively. EEG(t) denotes the uncorrupted EEG at time *t*. The regression coefficient $\beta_{g}$ measures the effect of EOG on eeg(t) at time (t˘g).

For example, to remove the effect of an artifact signal *Y* from an EEG signal *X*, we compute the regression correlation between the two signals. This denoising function allows us to successfully eliminate the artifact signal from the EEG. Algorithm 2 illustrates the general procedure.

However, the main concern raised about the regression method is bidirectional contamination [55]. For example, EOG recordings may include neural potential along with ocular potential [57,58]. Therefore, removing EOG activity from the EEG signal requires subtracting a portion of the relevant EEG signal from each recording. Furthermore, one of the challenges of regression techniques is that they may not be effective in dealing with other artifacts, such as EMG artifacts [59], due to the lack of clear reference channels. With the emergence of potentially more efficient algorithms such as principal component analysis (PCA) and independent component analysis (ICA) [60,61], the regression method is no longer the default choice for removing artifacts from an EEG caused by EOG or ECG signals.
**Algorithm** **2** Regression-based denoising of EEG signals**Input:**
EEG signal *X*, artifact signal *Y*
**Output:** Clean EEG signal *Z*
 **function**
Regression (X,Y)
    Calculate regression coefficients between *X* and *Y*
    Remove artifact from EEG signal    **return** Clean EEG signal *Z***end function**

#### 2.2.2. Blind Source Separation

Blind Source Separation (BSS) is a technique that separates source signals from a mixed signal without prior knowledge of the original signals. In the first step, the observed EEG is decomposed into its sources using the BSS algorithm. The noise sources are then identified and eliminated while preserving the sources of brain activity. BSS techniques are commonly used to denoise EEG signals [62]. The following techniques are described below.

Independent Component Analysis: Various types of ICA algorithms have been proposed in different papers, making it one of the key blind source separation techniques in biomedical engineering. ICA is capable of extracting statistically independent sources from a collection of recorded signals [63]. In general, denoising EEG signals using ICA can be expressed as follows [64]:

(2) X=AS

where the given equation involves three matrices: *X*, *A*, and *S*. Matrix *X* contains EEG data, matrix *A* represents the linear mixing of various sources (e.g., EEG and artifact sources), and matrix *S* consists of independent components, such as brain and artifact sources. After obtaining the independent components, they can be visually examined to identify any artifacts such as eye blinks and muscle activity. The EEG signals can be denoised by removing these components. Algorithm 3 presents the denoising EEG signals based on the ICA method.
**Algorithm** **3** ICA based denoising of EEG signals**Input:** *X*: EEG data matrix**Input:** n_components: number of independent components to estimate**Output:** *S*: matrix of independent components**Output:** *A*: estimated demixing matrix
    Center and whiten the *X*.    Initialize *A* randomly.    **repeat**        Update *A* by exploiting non-Gaussianity of independent sources.    **until** convergence    Compute *S* from *A* and *X*.    Identify artifact components in *S*.    Remove artifact components from *S*.    Reconstruct cleaned data from *S*.    **return** *S*, *A*

Due to the significant overlap between EEG signals and EMG artifacts in both spatial and temporal domains, conventional ICA algorithms often struggle to separate all EMG artifacts and generate a set of independent components. To address this problem, Li et al. introduced an improved ICA model called EMG removal by adding sources of EMG (ERASE) [65]. Specifically, EMG reference artifacts were involved from the head and neck muscles as input to the ICA, which increased the power of the EMG artifacts in a few independent components, resulting in a more accurate separation. In comparative tests, the ERASE algorithm removed an average of 26% more EMG artifacts from EEG signals than the traditional ICA algorithm. In [66], a new denoising framework was devised and merges ICA with the continuous wavelet transform (CWT). CWT and the K-means algorithm are used to map the detected blink artifact. EEG information is then preserved while denoising through ICA [66].

Principal Component Analysis: PCA, which stands for principal component analysis, is an easy-to-use data reduction technique that uses the principle of orthogonality to eliminate artifacts [54]. Through the utilization of PCA dimensionality reduction, it is possible to eliminate the presence of noise, represented by small eigenvalues, within the data. This process results in a partial denoising effect. Typically, given EEG data *X*, the main goal of PCA is to solve this equation:

(3) $$XX^{T}\omega_{i}=\lambda_{i}\omega_{i}$$

where the symbol λ denotes the eigenvalue, while ω represents the eigenvector. The technique for reducing the number of dimensions in a dataset is accomplished by decomposing the eigenvalues of the matrix $XX^{T}$. The resulting eigenvalues are then sorted and the top d values are selected to serve as a projection matrix. Subsequently, the projection matrix can be used to transform the EEG data denoted by *D* into new EEG data represented by $D^{*}=W^{*T}D$, while minimizing the presence of noise. The pseudocode for PCA is presented in Algorithm 4.
**Algorithm** **4** Typical principal component analysis**Input**: EEG data $D=\{x_{1},x_{2},...,x_{n}\}$, low-dimensional space dimension *d*.**Output**: Projection matrix $W^{*}=\left(w_{1},w_{2},...,w_{d}\right)$. **procedure** PCA(D):
     Sample centering $x_{i}\leftarrow x_{i}-\frac{1}{m}\sum_{i=1}^{m}x_{i}$.     Calculate $XX^{T}$.     Eigenvalue decomposition for $XX^{T}$.     Select the largest d eigenvalues.     $W^{*}=\left(w_{1},w_{2},...,w_{d}\right)$.     New EEG data $D^{*}=W^{*T}D$.     **Return**: $D^{*}$.

In recent times, there has been an increasing focus on utilizing principal component analysis in conjunction with other techniques to achieve EEG denoising. Patel et al. have demonstrated the effectiveness of combining ensemble empirical mode decomposition (EEMD) with PCA to efficiently detect and suppress artifacts in single-channel EEG data. This method can automatically detect and suppress eye artifacts after correct selection of the detection threshold [67]. In [68], a learning model based on PCA and semi-supervised support vector machine (SVM) is proposed. The model first preprocesses the EEG and uses PCA to reduce its dimensionality. After obtaining a set of optimal channel subsets, a semi-supervised classification model based on SVM is designed. This model determines the relationship between labeled data and unlabeled data by calculating the Euclidean distance between them, and then extracts features to identify them. The experimental results indicate that the method can achieve 84.3% correct classification results with only 40% labeled data, suggesting its potential in scenarios where only a small amount of labeled data is available [68].

#### 2.2.3. Canonical Correlation Analysis

In the context of EEG signals contaminated with muscle artifacts, canonical correlation analysis (CCA) is generally more effective than ICA [69]. Due to the relatively lower autocorrelation of muscular artifacts compared to brain activity, it is feasible to employ canonical correlation analysis (CCA) as a means of distinguishing between muscle activity and brain activity [70]. The common CCA formula used for denoising EEG signal is expressed as follows [71]:

(4) $$\max_{u}\frac{u^{T}R_{xy}v}{\sqrt{u^{T}R_{xx}u}\sqrt{v^{T}R_{yy}v}}$$

where the variables *u* and *v* represent canonical variates, which are linear combinations of channels. The EEG data and artifact data have covariance matrices denoted by $R_{xx}$ and $R_{yy}$, respectively. The cross-covariance matrix between the EEG data and artifact data is represented by $R_{xy}$. After obtaining the canonical variates, they can be used to eliminate artifacts from the EEG data by subtraction. The denoising steps based on CCA are presented in Algorithm 5.
**Algorithm** **5** CCA based denoising of EEG signal [72]**Input:** *X*: EEG data matrix**Input:** *Y*: matrix of auxiliary variables (e.g., EOG or ECG data)**Output:** *Z*: matrix of cleaned EEG data
Center and whiten the *X*.Initialize the weight matrices *A* and *B* randomly.**repeat**    Compute the canonical weights $w_{a}$ by maximizing the correlation between *X* and *Y* with respect to *A*. ▹$w_{a}$: weights used to linearly combine the EEG signal for one component    Compute the canonical weights $w_{b}$ by maximizing the correlation between *X* and *Y* with respect to *B*.▹$w_{b}$:weights used to linearly combine the auxiliary signal for one component    Update the weight matrices *A* and *B*.**until** convergenceCompute the cleaned data as $Z=A^{T}X$.**return** *Z*

In [69], P Sheoran et al. proposed a new algorithm that combined CCA and noise adjusted principal component transform (NAPCT) to eliminate noise in EEG data. Using CCA to estimate the noise covariance matrix and NAPCT to remove artifact components, the algorithm achieved this without human intervention [69]. Another study introduced an unsupervised automated eye artifact recognition and removal algorithm [73]. This algorithm used CCA to extract neural signals from data and used a multi-channel Wiener filter (MWF) to adaptively eliminate eye artifacts from multi-channel EEG data [73].

#### 2.2.4. Wavelet Transform

The signals recorded by EEG devices often contain irregularities. To analyze these non-stationary signals, the wavelet transform (WT) is a widely used method [74,75]. The conventional approach of WT divides the EEG signal into wavelet components. Components that contain artifacts are identified and removed, leaving only clean components. These clean components are then used to reconstruct a purified signal [76].

The WT is generally classified into two categories: discrete wavelet transform (DWT) and continuous wavelet transform (CWT) [77]. Given the continuous nature of the EEG signal, our attention is directed towards the DWT transformation. The DWT can be expressed in mathematical notation:

(5) $$\begin{array}{c} DWT\left(m,n\right)=\int_{-\infty }^{+\infty }x\left(t\right)\psi_{m,n}\left(t\right)dt \end{array}$$

where the variables “m” and “n” represent the scaling and translation factors, respectively.

The process of discrete wavelet analysis involves the decomposition of x(t) into various scales:

(6) $$x\left(t\right)=\sum_{j=1}^{K}\sum_{k=-\infty }^{\infty }d_{j}\left(k\right)\psi_{j,k}\left(t\right)+\sum_{k=-\infty }^{\infty }a_{K}\left(k\right)\phi_{K,k}\left(t\right)$$

where the given equation involves discrete analysis wavelets represented by $\psi_{j,k}\left(t\right)$ and discrete scaling functions represented by $\phi_{K,k}\left(t\right)$. The variable $d_{j}\left(k\right)$ denotes the detailed signals or wavelet coefficients at a scale of $2^{j}$, while $a_{K}\left(k\right)$ represents the approximated signal or scaling coefficients at a scale of $2^{K}$.

DWT is a method of transforming time domain EEG signals without redundancy, which is useful in removing artifacts. To accomplish this, the signal undergoes a series of low-pass and high-pass filters to obtain approximate and detailed coefficients. This process is repeated until the desired frequency is achieved. In Algorithm 6, we present the pseudocode for DWT. However, DWT has the drawback of lacking translation invariance. The stationary wavelet transform (SWT) can overcome this issue, but it has its own limitations, such as redundancy and slow speed [78].
**Algorithm** **6** DWT based denoising of EEG signal [79]**Input:** *X*: EEG data matrix (rows represent the EEG channels)**Output:** *Y*: matrix of cleaned data
Set the wavelet basis and level of decomposition**for** each channel *c* in *X* **do**
    Compute the DWT coefficients of *c* at each level using the fatigue wavelet basis.    Identify the approximation coefficients at the desired level as the artifact-free signal.    Threshold the detail coefficients using a soft or hard thresholding technique.    Reconstruct the cleaned signal by inverse DWT using the modified coefficients.    Store the cleaned signal in the corresponding row of *Y*.**end for****return** *Y*

WT alone may not be sufficient to address all the issues associated with EEG signal denoising, as it can result in information loss and signal reconstruction problems. Therefore, the combination of the wavelet transform with other techniques to improve the denoising process has been explored. For instance, the authors of [80] applied ICA to separate signals based on WT and found that this combination was effective in removing EMG noise and ECG artifacts from EEG signals. In addition, notch filters can be used in conjunction with WT to address the issue of overlapping spectra/frequencies between EEG signals and artifacts. In [81], an adaptive threshold for wavelet coefficients was used to eliminate frequent ocular artifacts (OA) and a 50 Hz IIR notch filter to reduce artifacts and noise while preserving the original brain signals.

#### 2.2.5. Empirical Mode Decomposition

Empirical mode decomposition (EMD) is a technique for analyzing non-stationary and nonlinear signals that offers several desirable properties. EMD leverages the signal extreme points to decompose a signal into a set of intrinsic mode functions (IMFs) and a monotonic residual, which can be expressed using the following formula [82]:

(7) $$x\left(t\right)=\sum_{i=1}^{n}C_{i}\left(t\right)+r_{N}\left(t\right)$$

where $C_{i}$ represents an IMF and $r_{N}$ represents the monotonic residual.

The IMFs are capable of capturing the fundamental oscillatory components at various frequencies, which facilitates the differentiation between artifacts and the intended EEG signal. To obtain the high-frequency content of a signal, including any artifacts, as a detail component, one can subtract the envelope from the input signal. The envelope represents the smooth curve that passes through the local maxima and minima of the signal. This technique enables the elimination of undesired artifacts while retaining the original EEG signal. The reconstructed signal is obtained by adding up the detailed components after cleaning. More details are listed in Algorithm 7.
**Algorithm** **7** Empirical Mode Decomposition (EMD) for EEG Artifact Removal [82]**Input:** *X*: EEG data matrix**Output:** *Y*: matrix of cleaned data
Set the stopping criterion and number of maximum iterations.**for** each channel *c* in *X* **do**
    Initialize $d_{0}=c$, k=1                                  ▹$d_{k}$: signal at iteration *k*    **repeat**
        Find the local maxima and minima of $d_{k-1}$.        Compute the envelope by interpolating the maxima and minima.        Subtract the envelope from $d_{k-1}$ to obtain the detail component $h_{k}$.     ▹*h*: detail component        Update $d_{k}=d_{k-1}-h_{k}$.        Increment *k*.
    **until** stopping criterion or maximum iterations are reached.    Compute the reconstructed signal as $r_{c}=\sum_{i=1}^{k}h_{i}$.             ▹$r_{c}$: reconstructed signal for channel *c*    Store the cleaned signal in the corresponding row of *Y*.
**end for****return** *Y*

One of the advantages of EMD is its ability to extract local amplitude, phase, and frequency content from the resulting components. EMD is also adaptive and efficient and, when combined with other techniques, it can lead to new advancements in the denoising of EEG signals. For instance, combining ensemble empirical mode decomposition (EEMD) with the CCA technique led to feasible results. Specifically, EEMD generates a large number of IMFs, increasing the number of channels available for ICA. This method leverages interchannel information and addresses the challenging problem of CCA in dealing with EEG data with low signal-to-noise ratio (SNR) and complex contamination [83].

EMD is highly sensitive to spike noise because of its reliance on extreme signal point features for IMF decomposition. This sensitivity can lead to the mode-splitting effect, which can seriously affect the removal of EOG artifacts. To address this issue, the multivariate adaptive moving average–empirical mode decomposition (MAMA-EMD) based method extracts peaks into the first IMF to improve the accuracy of subsequent IMF screening and alleviate the mode-splitting effect [84]. However, MAMA-EMD may not achieve optimal results in separating spikes when the pulse has two or more consecutive spike points. To address this limitation, a new version of MAMA-EMD is proposed by supplementing the minimum arc length criterion. This approach effectively eliminates the influence of multi-point spikes on the screening process [85].

In recent years, various classic and commonly used denoising methods have been combined to achieve better signal denoising in different situations. Some examples of these methods or their combinations that have been used for denoising purposes are summarized in Table 2.

### 2.3. Evaluation Criteria for Denoising

Various metrics, such as mean squared error (MSE), root mean squared error (RMSE), signal-to-noise ratio (SNR), and percentage root mean square difference (PRD), are commonly used to assess the effectiveness of EEG signal denoising [86]. MSE is frequently employed to evaluate the similarity between the initial EEG signal and the noise-reduced signal. The RMSE is mathematically defined as the square root of the MSE. The SNR is a metric used to compare the magnitudes of the signal and noise power. The PRD is used to measure the degree of similarity between the original and noise-reduced signals, with a lower PRD indicating a higher degree of similarity between the two signals. These metrics can be defined as:

(8) $$\begin{array}{c} \mathrm{MSE}=\frac{1}{n}\sum_{i=1}^{n}{\left(y_{i}-{\hat{y}}_{i}\right)}^{2},\mathrm{RMSE}=\sqrt{\frac{1}{n}\sum_{i=1}^{n}{\left(y_{i}-{\hat{y}}_{i}\right)}^{2}}\\ \mathrm{SNR}=10\log_{10}\left(\frac{\sum_{i=1}^{n}y_{i}^{2}}{\sum_{i=1}^{n}{\left(y_{i}-{\hat{y}}_{i}\right)}^{2}}\right),\mathrm{PRD}=\frac{100\%}{n}\sum_{i=1}^{n}\frac{|y_{i}-{\hat{y}}_{i}|}{|y_{i}|} \end{array}$$

where $y_{i}$ typically refers to the true EEG signal value at time point *i*, ${\hat{y}}_{i}$ represents the denoised value of the EEG signal at time point *i*, and *n* is the total number of values.

### 2.4. Feature Engineering

Enhancing feature engineering can improve the accuracy of predictions made on raw data by transforming them into more expressive features. Extracting the available features from the processing of EEG signals is a complex task that typically requires multiple human experts with specialized knowledge. Machine learning techniques, such as deep neural networks and adversarial generative networks, have allowed the automated extraction of features from EEG signals. However, the interpretability problem of deep learning is deeply criticized. Recently, the advancement of explainable AI (XAI) methods [87] has aimed at improving the interpretability of deep learning models. For example, XAI based Smooth-Grad [88] is used to perform EEG based emotion recognition [89], seizure detection [90], and other applications. Using XAI methods, it allows eliminating the need for manual selection by human experts. In [91], the authors proposed a method to achieve high-resolution assessment of neural activity using deep networks, involving the implementation of relevant layer propagation. They used an adversarial generation network to produce EEG signals [92]. However, efficient feature engineering methodologies can help machine learning models acquire the fundamental features implicit in EEG more easily. Therefore, we examine the main feature engineering methodologies used in EEG [91,92,93]. Commonly utilized conventional signal processing techniques in various research studies on EEG signal processing include time–frequency analysis, high-order spectrum analysis, and nonlinear dynamics analysis.

#### 2.4.1. Time–Frequency Analysis

The primary purpose of time–frequency analysis is to establish a link between the time and frequency domains. This involves analyzing and processing signals in both domains to extract relevant features. The most commonly used methods for analyzing stationary signals include analysis of variance, waveform parameter analysis, wave identification, histogram analysis, correlation analysis, and others. These methods are often applied in the diagnosis of diseases [94]. For example, time–frequency analysis is used to map EEG signals in the time and frequency domains. By dividing the signal data into windows and scoring them, it is possible to identify epilepsy signals in the time–frequency domain [94]. Another example is related to the detection of peak features in EEG signals [95]. Specifically, the signal is denoised using SVD and then the peak is detected, which yields superior results [95]. In [96], a high resolution non-parametric time–frequency method is proposed to analyze EEG signals that uses CNN to optimize the Wigner–Ville distribution as an input without parameters, showing its clear superiority over other methods.

#### 2.4.2. High-Order Spectral Analysis

Although the time domain analysis method falls short in analyzing high-order information and providing complete signal feature results, the high-order spectral analysis method can effectively address this limitation [97,98]. With its ability to map specific information more effectively in EEG signal processing, higher-order spectral analysis demonstrates significant superiority [97]. It is capable of suppressing Gaussian noise and producing spectral structures that reflect more information, as evidenced by simulation experiments on more than 200 EEG samples conducted by some authors [97]. The resulting spectral lines are flatter, with less noise and smoother contours [97]. To identify the nonlinearity and high dimensionality present in epileptic signals, the principal component features are extracted using PCA on the 15 high-order spectra (HOS) features extracted from the EEG data [99]. Furthermore, in [100], they use HOS to analyze EEG signals in the field of neuro-marketing. According to the study findings, the proposed model, using SVM with Gaussian kernel, achieved an average accuracy of 73.24% across all users. Moreover, HOS features were used to access participants who were in a typical emotional state but not exhibiting any motor movements [101]. Experiments demonstrated that the method achieved an average accuracy of 95.7%.

#### 2.4.3. Nonlinear Dynamic Analysis

Previous work has shown that the traditional linear analysis method is inadequate for accurately evaluating the dynamic structure of EEG signals and as a result it cannot reveal the essential characteristics of brain activity. However, the use of nonlinear dynamic methods to extract and analyze EEG signals has provided a new approach to further study the process and characteristics of human brain activity [102].

Much literature in this field uses nonlinear techniques, such as the Lyapunov exponent, complexity measures, and fractal dimension, to analyze EEG signals. For example, a researcher used the correlation dimension of nonlinear dynamics and the Lyapunov index to extract characteristics of high-frequency EEG from elderly and young subjects during various activities, including silent eye closure, silent eye-opening, and N-back letter memory events. They then performed a statistical analysis on the resulting eigenvalues to compare the differences between the two groups of eigenvalues [103].

The features of the EEG signals were effectively extracted by various indicators such as the Hurst index, the Lyapunov index, the sample entropy, and the wavelet entropy [104]. Furthermore, an adaptive Lempel–Ziv complexity algorithm was proposed and utilized to measure the complexity of EEG signals, which was capable of identifying emotions [105]. By comparing the complexity values of the traditional Lempel–Ziv–Welch compression algorithm (LZC) and the multiscale and adaptive LZC algorithm on the corresponding electrodes under three emotional states, it was discovered that the adaptive LZC algorithm could effectively distinguish between the three different emotional states. The algorithmic processes of permutation entropy and sample entropy were briefly introduced and their respective advantages and disadvantages were analyzed in detail [106]. A new algorithm called equal symbolic entropy (ESE) was proposed, and its effectiveness in terms of accuracy and efficiency was verified through simulation. It was applied to analyze emotional human EEG signals from an experimental group [107].

Furthermore, due to the intricate nature of the EEG signal, traditional linear techniques face significant challenges when analyzing it, leading to the application of nonlinear dynamics methods in the analysis of EEG signals. Various features such as the correlation dimension, the fractal dimension, the complexity, the approximate entropy, and others have been explored in the literature, along with the corresponding methods for extracting these features from EEG signals. These findings provide evidence supporting the scientific basis of utilizing nonlinear dynamics for EEG signal analysis [108].

### 2.5. EEG Based Classifications

EEG signal classification is a fundamental task in the analysis of brain function, which can be considered as one-dimensional biomedical signal processing [109,110,111]. Various processing methods can be employed to classify EEG signals, including statistics, machine learning (deep learning), and other techniques. In this section, we will focus on the classification methods used in various fields of EEG application, with particular emphasis on machine learning-based approaches. Furthermore, we have listed the commonly used EEG datasets in Table 3.

#### 2.5.1. Traditional Classification Method

The classification of brain signals through the application of ML techniques mainly involves the use of supervised and unsupervised learning methods. These methods include naive Bayesian (NB), decision tree (DT), K-nearest neighbor (KNN), support vector machine (SVM), and random forest (RF), among others. Supervised learning is related to using input and anticipated output data to develop predictive models that cater to classification and regression. On the other hand, unsupervised learning involves proposing a prediction model that uses input data for clustering and dimension reduction [124]. Supervised learning yields higher accuracy than unsupervised learning when using classifiers such as SVM or KNN. The precision of a solitary classification technique is restricted to particular use cases. Hence, multimodal integration algorithms are commonly employed in studies to improve the overall accuracy of classification. The increasing complexity of algorithms may result in bias and affect their accuracy. Previous research has used machine learning methods to examine EEG signals for the identification of diseases (e.g., epilepsy, depression, stroke) and rehabilitation interventions (e.g., motion imagination). Specifically, Table 4 summarizes literature examples related to epilepsy, motion imagination, depression, and stroke. In general, the results in Table 4, suggest that the SVM model is an important classifier model for EEG signals.

We also summarize many publications in Table 5, related to EEG signals to evaluate many other tasks. For example, ANN and KNN classifiers are used to classify the EEG signals into epileptic and non-epileptic classes. The results showed that the combination of statistical parameters and classifiers achieved an accuracy of 95.9% for ANN and 92.4% for KNN, indicating the effectiveness of the proposed method in detecting epilepsy using EEG signals [125]. EEG with an SVM classifier based on nonlinear feature extraction is used to improve the recognition rate of epileptic brain signals. They decomposed the EEG signal into different frequency bands through a four-layer WT and the approximate entropy (ApEn) value of the wavelet coefficients in each frequency band was used as the eigenvector input. The correct recognition rates for normal and epileptic EEG signals were 98.3% and 95.6%, respectively, which outperformed other similar algorithms [126]. Furthermore, the results showed that RF performed better than AdaBoost and KNN in eliminating error detection [127].

Furthermore, there is extensive research on classifying EEG signals in motor imagery (MI), a process that involves imagining actions without physically executing them. This research is important in helping patients who have lost motor function in recovery. In [128], it proposed to add Gaussian noise to EEG signals to improve recognition rates and perform binary classification (left hand and right hand) MI tasks. The KNN algorithm achieved a maximum average classification accuracy of 88.57% [129]; the LDA algorithm was used to reduce the dimension of the feature data in the classification of MI EEG signals. When combined with KNN, they achieved average classification accuracies of 67.5% and 84.62%, respectively. This improved classification accuracy and speed, demonstrating the algorithm’s advantages in lower-limb MI classification. In [130], the mixed feature majority vote classifier is used to recognize MI EEG signals. They selected the combined feature as the input of the classifier and used most voting classifiers to combine SVM, LDA, and ANN. The accuracy of performance measurement reached 85.36%, indicating that the proposed system outperformed the conventional machine learning EEG recognition classifier. In [135], they expanded the use of BCI to include motor imagery and presented a framework that used augmented covariance extracted from an autoregressive model for classification purposes.

**Table 5:** Summary of various EEG studies in a variety of applications.

Ref.	Domain	Proposed Method	Conclusion
Alharbi and Alotaibi [136]	GD	Proposed Hamming window bandpass FIR filter model for automatic gender identification using classifiers	The RF classifier based on negative emotion EEG signal had the lowest error percentage
Parmar [137]	Dyslexia	Evaluated the performance of the nonlinear kernel of SVM Gaussian kernel (RBF), polynomial kernel, and sigmoid kernel	The maximum accuracy rate of RBF kernel for nonverbal stimuli reached 62.4%, with good performance
Ling and Aihua [138]	BCI	Constructed a multi-class SVM classifier combining DT and SVM	The highest classification accuracy reached 80.8%
Hossain [139]	CR	Used a new method to decode English letters directly from EEG signals	The accuracy of KNN was 81.6% better than SVM and NB in the classification of EEG signals with different letters
Padayatty and K [140]	Schizophrenia	Design of a suitable classifier to distinguish SZ EEG signals from HC EEG signals	SVM provided the best performance with a correct classification rate of 90.14% for SZ and an overall accuracy rate of 89.58% for the EEG data considered
Yuehua and Jinxiang [141]	Vertigo state	Classification of vertigo states based on machine learning and EEG signal analysis	The RF model had the best classification, with an accuracy rate of 82.5%
Shuyi [142]	Alcoholic	Used NTFT and k-cross validation method	KNN classifier achieved good results in average accuracy which were up to 99%
Satyanarayana [143]	Emotions	An SVM emotion classifier based on EEG	The results obtained were 83% accurate in detecting emotions

GD: Gender detection; FIR: Finite impulse response; RBF: Radial basis function; BCI: Brain–computer interface; SZ: Schizophrenia; NTFT: Normal time–frequency transformation; HC: Healthy control; CR: Character recognition; RBF: Radial basis function; ICH: Intracerebral hemorrhage; HRV: Heart rate variability; RESP: Respiratory rate; DFA: Declined fluctuation analysis; (see other abbreviations noted in Table 4).

The advancement in EEG classification models has led to new possibilities in detecting depression, a mental disorder that affects a significant portion of the global population. To recognize depression based on EEG signals, some researchers have utilized the tree model’s feature selection algorithm to establish a depression recognition model. It is clear from Table 5 that research on the use of traditional ML algorithms to classify EEG signals is still ongoing and is expanding. EEG signals are complex patterns of electrical activity in the brain, and accurately classifying them can be crucial to understanding various neurological conditions and cognitive processes.

Several research studies have utilized entropy measurement and statistical features of EEG signals in gender detection to enhance its accuracy. To obtain EEG data on negative and positive emotions for training and testing, a finite impulse response (FIR) filter model is commonly employed. Decision trees, random forests, and multi-layer perceptron are popularly used to predict gender from the obtained data. The findings suggest that the random forest classifier performs best with the EEG of negative emotions, and investigates the effect of excluding individual and multiple electrodes from the EEG data on the system performance [136].

According to a study in [140], EEG can be used to detect neurophysiological changes associated with schizophrenia. In another study, external vestibular electrical stimulation was used to induce vertigo symptoms and EEG features were extracted using a wavelet decomposition algorithm. The extracted features were then classified into different levels of vertigo using logical regression, SVM, backpropagation, and RF classifiers. The RF model demonstrated the highest accuracy of 82.5% [141]. Additionally, a regular time–frequency transform technique was applied in [142] to predict EEG signals and evaluate individuals with alcoholism at different stages. Furthermore, SVM was used for emotion recognition based on EEG with large datasets [143].

In addition to the previous points, EEG signals can also be used for fatigue detection. In [144], an advanced machine learning method was proposed to use EEG signals to detect driver fatigue and alert the driver as early as possible to prevent potential risks while driving. This method is based on a flexible analytic wavelet transform. In [145], they presented forehead EEG in combination with machine vision for detecting fatigue in real-time. Experiments demonstrated that the proposed method could achieve significant performance. In the field of aviation, in [146], they proposed using EEG to discriminate aircraft pilot cognitive workload during flight, which achieved an accuracy of 91.67% in classification tasks. Furthermore, in the maritime field, an approach was proposed for assessing mental fatigue based on EEG frequency bands [147]. This approach was intended for demanding maritime operations. The approach was tested in a realistic vessel simulator and the results indicated that it could detect increased mental fatigue levels. EEG can also be useful in the work context. For example, in [148], they proposed a measure that uses implicit EEG signals to predict workers’ experience as a proxy for their ability to recognize hazards. This leads to further improvement in the investigation of how we can derive greater benefits from EEG signals.

#### 2.5.2. Deep Learning

**Figure 4:**
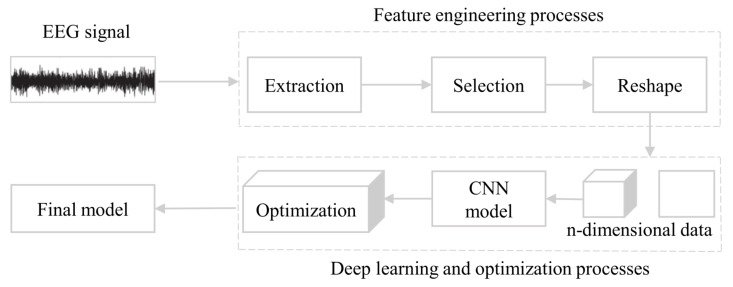
The pipeline of DL models for EEG classification [149]. The pipeline starts with feature engineering applied to the EEG signal. The processed data is then transformed into 2D or 3D format, which serves as input to a convolutional neural network (CNN) model. Finally, the CNN model undergoes training and optimization, leading to the identification of an optimal model as the final result of the pipeline.

Although the structure of deep learning models is more complex compared to traditional ML, they offer greater advantages in classifying and predicting EEG signals. Several researchers have utilized deep learning models to analyze EEG signals for disease detection such as Alzheimer’s disease, epilepsy, ischemic stroke, etc. and to predict the progression of these diseases. A summary of these methods is discussed and reported in Table 6. In Figure 4, we provide a clear view of how DL can be used for EEG data classification [149]. Initially, the EEG data are subjected to denoising and subsequent feature engineering. Afterwards, the processed data is converted into two-dimensional (2D) or three-dimensional (3D) data, which serves as input to the CNN model. Finally, the CNN model is subjected to training and optimization, and the optimal model is selected as the final model.

Continuous development in stroke research has been achieved through the use of deep learning models based on EEG signals. To prevent stroke effectively, a deep learning-based stroke evaluation model has been used. This model extracts mel frequency cepstral coefficient (MFCC) features and inputs them into a CNN, which achieves 22.86% higher accuracy compared to logistic regression [156]. To extract more information from signals passing through multiple convolution layers, hidden layers, and filters effectively, another studies utilized VGG-16 and Resnet-50 models for stroke detection, resulting in a model accuracy of 90% [157].

In order to detect MDD, EEG data has been analyzed using the DeprNet model [158,159]. Additionally, AlexNet [165] and GoogleNet [166] are utilized to identify sleep disturbances from EEG signals through visual recognition tasks. Three categories of EEG signals were analyzed, namely epilepsy, normal EEG, and sleep disorders. Results indicate that AlexNet outperforms GoogleNet in detecting sleep disorders, achieving an accuracy of 93.33% [160]. In a separate study, a combination of CNN and RNN is employed to classify sleep stages using the EEG channel Fp1/Fp2, achieving an accuracy of 79.7% [161]. The studies demonstrate the potential of deep learning algorithms in enhancing the automatic classification of sleep stages based on EEG signals.

Using the EEG, a study investigated an end-to-end deep neural network for accurately classifying drivers’ cognitive workload with high accuracy [163]. Additionally, a recent study proposed a categorization system for driver fatigue that employs EEG signals in conjunction with machine learning and deep learning algorithms. The results demonstrated a significant level of precision in distinguishing between various fatigue states [164].

In [152], an approach aims to minimize human intervention while ensuring that all the necessary components for EEG analysis are integrated in a logical and comprehensible way. The model demonstrates significant performance in detecting Alzheimer’s disease early, as indicated by its high ROC-AUC score of 0.9 [152].

The proposal utilized a deep learning method that employed EEG signals recorded by the Muse EEG headband for performing emotion recognition tasks.

In [167], a proposal for an EEG-based brain–computer interface (BCI) was presented. It uses a deep learning method that employed EEG signals recorded by the Muse EEG headband for performing emotion recognition tasks. Furthermore, in [168], a new lightweight multidimensional attention network was proposed to address issues related to poor generalization across datasets, high predicting volatility, and low model interpretability. The method led to an enhanced classification performance in various BCI tasks.

## 3. Future Directions and Common Challenges

ML methods often face challenges related to data, as they may require larger datasets than traditional methods to achieve similar performance. EEG data collection can be complex and challenging, and continuous improvement of ML models is necessary to fit older processing pipelines for better performance or to reduce the required amount of data [169]. However, deep learning has simplified the EEG signal processing pipeline, making it an end-to-end task [93]. Furthermore, deep learning has facilitated new research avenues, such as generating images from EEG signals and transfer learning between different fields [169,170,171,172]. Overall, EEG signals are a valuable source of information for understanding brain activity, and both traditional and ML methods offer unique benefits and challenges for processing these signals.

The matter of data heterogeneity in EEG research arises due to variations in the acquisition devices employed across different datasets. Domain adaptation (DA) is a technique employed to address the challenge of data heterogeneity by leveraging similar domain data to reduce the data discrepancy [173]. The utilization of DA can serve as a means of alleviating this particular issue. The authors of [174] introduced a multi-modal domain adaptive variational autoencoder approach to enhance the performance of emotion recognition tasks based on EEG data.

Additionally, the implementation of data privacy laws such as the General Data Protection Regulation (GDPR) [175] can make it difficult to directly access source data for training due to personally identifiable information from patients present in the EEG data. Federated learning (FL) is a learning method that involves training multiple local models and then obtaining a global model by aggregating these models globally without sharing raw data [176]. In [177], the authors proposed a transferable FL technique to perform EEG classification tasks. The experimental results show that the approach utilized by the researchers can achieve an average accuracy of 91.10% using the Sleep-EDF dataset [178].

The field of EEG research offers access to several databases that contain EEG data. These databases contain recordings of electrical brain activity obtained from multiple electrodes and can be used to investigate a variety of research questions. For example, some databases include EEG recordings of seizures from their onset to their end, while others contain EEG data of participants performing different types of movements. Additionally, some databases provide auditory-evoked EEG recordings, which capture the brain’s response to sound stimuli. Researchers can use these datasets to advance their understanding of brain function and behavior.

As previously mentioned, EEG signal processing presents several challenges that are summarized as follows.

- EEG data often contain noise and artifacts from various sources, such as muscle movements, eye blinks, electrocardiogram signals, and electrical interference. These unwanted components can significantly affect the quality of EEG signals.
- EEG signals are non-stationary, meaning that their statistical properties change over time, making it difficult to analyze them using traditional methods. This characteristic requires specialized techniques to capture the time-varying nature of EEG signals.
- EEG electrodes record signals originating from multiple sources in the brain, which can result in a phenomenon called volume conduction. The superposition of signals from multiple sources makes it challenging to locate the exact source of specific signals.
- The EEG signal acquisition measures the potential difference between the acting electrode and the reference electrode. This leads to the problem of electrode reference. The data obtained can vary depending on the selection of the reference electrode. Selecting the best point for the reference electrode can be a challenging task.
- One of the challenges in EEG-based deep learning models is their interpretability. If we can interpret the deep learning model accurately, patients may have more trust in the machine learning diagnosis than in the diagnosis given by a doctor [89].
- EEG signals vary between individuals due to differences in skull thickness, conductivity, and brain structure, making it difficult to compare data between subjects. Specialized analysis methods must be employed to account for individual differences while comparing EEG signals.
- Interpreting EEG signals requires expertise in both neuroscience and signal processing, as they are indirect measures of neural activity. Proper analysis with different machine learning algorithms might help to decode specific features of the signal that relate to cognitive or behavioral states.

Addressing these challenges requires the development of new methods that can handle these unique features of EEG signals, including denoising, source localization, improved electrode configurations, and AI based on signal processing techniques.

## 4. Conclusions

This paper presents a comprehensive analysis of various techniques used for EEG preprocessing and feature extraction. We also discuss EEG acquisition methods and summarize signal denoising processes, including regression, blind source separation, wavelet transform, and empirical mode decomposition. Our study focuses on time–frequency analysis, high-order spectral analysis, and nonlinear dynamic analysis, and their applications in EEG feature engineering. We observed that machine learning algorithms have the potential to achieve high accuracy in EEG classification, although the accuracy of classifiers varies. We also found that deep learning models exhibit a comparable accuracy in detecting seizures. To date, AI based algorithms have the potential to improve EEG analysis and diagnosis, leading to better patient outcomes.

## Figures and Tables

**Table 1:** Research on EEG applications.

Ref.	Prupose	Acquisition Method	Data Processing
Ma [42]	Recognize driver fatigue	Commercial Neuroscan system with 40 electrodes	Third-order Butterworth bandpass filter
Gamage [43]	Detect driver’s EEG to reduce traffic accidents	Evoke the emotions of the test driver with video and audio	EEGLAB Toolbox of Matlab
Shen [44]	Strengthen the depression recognition performance	Traditional 128-electrode mounted elastic cap and a wearable 3-electrode EEG collector	EEGLAB Toolbox of Matlab
Saedi [45]	Detect the working status of construction workers	Investigate mental and motor work	A mix of macro and micro scrutiny
Han [46]	Classification of eye state	EEG measured around the ear	Estimating classification accuracy using 3 CNN models
Pawuś and Paszkiel [47]	Use BCI to control the robot	Emotiv EPOC	Classic algorithms and the neural network
Chen [48]	EEG decoding	Obtained in the open world	Supervised deep learning
Pei [49]	PreG electrode in BCI	Obtained form PreG electrode	SSVEP-based BCI
Jemal [50]	Epileptic seizure prediction	Publicly available CHB-MIT dataset	Deep neural network model
Wen [51]	Evaluate spatial cognitive ability	From 7 subjects participating in the game	Coupling strength calculation
Li [52]	Emotion recognition	SJTU Emotion EEG Dataset	Experiment-level BN
Freismuth et al. [53]	Treatment and diagnosis of ADHD	Wearable EEG device	HiLCPS framework

CNN: Convolutional neural network; BCI: Brain–machine interface; BN: Batch normalization; ADHD: Attention deficit hyperactivity disorder; HiLCPS: Human-in-the-loop cyber-physical systems.

**Table 2:** Application of denoising method in EEG signal.

Ref.	Signal Processing Method	Conclusion
Li [65]	EMG reference artifacts of neck and head muscles	More precise EMG separation without manual intervention
Maddirala and Veluvolu [66]	CWT and K-means	It is suitable for situations with few EEG signal channels and can accurately separate artifacts
Patel [67]	Combining EEMD and PCA	Automatic detection and suppression of human eye artifacts can be achieved
Xie [68]	PCA with an SVM-based semi-supervised classification model	It is suitable for processing signals with a low signal-to-noise ratio and only a few labels, with high recognition accuracy and less training time
Sheoran and Saini [69]	Combining CCA and NAPCT	Artifact components are removed without manual intervention
Miao [73]	CCA and MWF	Eye artifacts can be adaptively removed from multi-channel EEG data without the need for a reference signal
Zhou and Gotman [80]	Wavelet transform	The combination of wavelet transform and ICA can effectively remove EMG and ECG artifacts in EEG signals
Tibdewal [81]	Use the adaptive threshold of wavelet coefficients	Effectively reduces artifacts and noise while preserving the original brain signal
Chen [83]	EEMD and CCA techniques	It can make good use of interchannel information and has a good artifact removal effect in the case of serious signal pollution
Yang [84]	Extract spikes to the first IMF	Can alleviate splitting effects, but not suitable for separating multipoint spikes
Li and Zhang [85]	EMD	It can eliminate the effect of multipoint spikes on IMF screening and better remove EOG artifacts

ICA: Independent component analysis; PCA: Principal component analysis; CCA: Canonical correlation analysis; WT: Wavelet transform; EMD: Empirical mode decomposition; CWT: Continuous wavelet transform; EEMD: Ensemble empirical mode decomposition; NAPCT: Noise adjusted principal component transform; MWF: Multi-channel Wiener filter.

**Table 3:** Summary of commonly used EEG signal analysis datasets.

Dataset	Sample (n)	Types	SF (Hz)
Zhang [112]	122	Object recognition	256
Koelstra [113]	32	Emotion analysis	128
Zheng and Lu [114]	15	Emotion recognition	200
Ang [115]	9	Emotion recognition	250
Tangermann [116]	9	BCI	250
Sajda [117]	9	BCI	100
Andrzejak [118]	10	Seizure detection	173.86
Shoeb [119]	23	Seizure detection/prediction	256
Detti [120]	14	Seizure detection/prediction	512
Zhang [121]	6	Mental workload	500
Venkatachalam [122]	5	MIC	150
Zhang [123]	64	EEG denoising	512

BCI: Brain computer interface; MIC: Motor imagery classification; SF: Sample frequency.

**Table 4:** Application of traditional classification method in EEG research.

Ref.	Domain	Propose Method	Conclusion
H and A. [125]	Epilepsy	Used KNN and ANN classifiers to predict seizures	For KNN classifier, HFD with sample entropy had the highest accuracy of about 98%
Ping [126]	Epilepsy	Created an SVM classifier based on nonlinear feature extraction	Successfully improved the correct recognition rate
Jamunadevi [127]	Epilepsy	Used RF for detection and evaluation	RF had better results in eliminating epilepsy error detection
Jiahui [128]	MI	Added Gaussian noise and performed binary classification	The maximum average classification accuracy of KNN classifier reached 88.57%
Jiaying [129]	MI	Created a lower extremity MI classification algorithm based on LDA+KNN.	The average classification accuracy of the two paradigms was 67.5% and 84.62%, respectively
Dongare and Padole [130]	MI	Created a majority voting classifier that combines SVM, LDA, and ANN	The accuracy of performance measurement was 85.36%
Ren [131]	Stroke	Adopted C4.5 decision tree	Constructed a DT model with 37 nodes
Huaiwen and Yin [132]	Stroke	Used ROC and AUC for model screening	The SVM model performed best as AUC = 1.000
Hanqi [133]	Stroke	Build model based on LASSO, DWI, PWI, and SVM	The accuracy of the combined model was 0.822, which was better than that of the single sequence model
Yong [134]	Stroke	Used CTA image collection set data and K-fold cross validation	The random forest model had the best prediction effect, with an accuracy of 94.9% and 90.8% in predicting new ischemic stroke

DWT: Discrete wavelet transform; SVM: Support vector machines; KNN: K-nearest neighbor; ANN: Artificial neural network; HFD: Higuchi fractal dimension; RF: Random forest; MI: Motor imagery; LDA: Linear discriminant analysis; FIR: Finite impulse response; SSA: Sparrow search algorithm; K-S: Kolmogorov–Smirnov; NB: Naive Bayesian; MF-DFA: Multifractal declined fluctuation analysis; DT: Decision tree; ROC: Receiver operating characteristic; AUC: Area under ROC curve; DWI: Diffusion weighted imaging; PWI: Perfusion weighted imaging; CTA: Computed tomographic arteriography.

**Table 6:** Application of the deep learning method in EEG research.

Ref.	Domain	Proposed Method	Conclusion
Morabito [150]	Alzheimer’s disease	A method was proposed to generate a suitable feature set using convolution and then use full connectivity to make predictions	The method achieved 80% classification accuracy in Alzheimer’s disease
Morabito [151]	Alzheimer’s disease	A deep learning processing system to reduce the dimensionality of the feature space	The system achieved nearly 90% classification accuracy in diagnosing Alzheimer’s disease
Kim [152]	Alzheimer’s disease	A novel end-to-end model designed for the purpose of low-cost and noninvasive diagnosis of brain disorders	Their method achieved a high ROC-AUC score of 0.9
Kunekar [153]	Epilepsy	A deep learning and multimodal fusion approach was proposed for the diagnosis of epilepsy	The method allowed for improved diagnostic accuracy and earlier prediction of seizures due to the continuous performance of the data
Sagga [154]	Epilepsy	Proposed a simple CNN model to identify epileptic seizures	The CNN model achieved 98% accuracy in seizure detection
Qing [155]	Epilepsy	Using neural network model to process one-dimensional time series and two-dimensional EEG image EEG data types to detect seizures	The classification accuracy of EfficientNetV2 model for epileptic EEG was 98.69%
Ouyu [156]	Ischemic stroke	A deep learning-based stroke evaluation model for stroke diagnosis	CNN was 22.86% more accurate than logistic regression
Kumar and Sengupta [157]	Ischemic stroke	Stroke detection using VGG-16 and Resnet-50 models	The accuracy of the model in predicting stroke reached 90%
Seal [158]	Depression	A CNN DeprNet was proposed for depression diagnosis	The accuracy of the results obtained in recording split and subjective split experiments was 99.37% and 91.4%, respectively
Rafiei [159]	Depression	Automatic detection of MDD Using EEG data and deep neural network architecture	The accuracy reached 91.67% when all 19 channels were used and 87.5% after the channels were reduced
Sudhakar [160]	Sleep	Alexnet and GoogleNet used EEG signals to detect sleep disorders	AlexNet was better at detecting sleep disorders with an accuracy of 93.33%
Leino [161]	Sleep	Combined CNN and RNN to determine the sleep stage of the EEG channel measured by AES	When considering all datasets, the highest automatic scoring accuracy was 79.7%
Kang and Hong [162]	Sleep	The optimized GoogleNet model was used to construct CNN automatic sleep stage classification in single channel EEG	The accuracy of the sleep state of the EEG F4 channel was the highest at 77.6%
Almogbel [163]	Cognitive	An end-to-end deep neural network could accommodate the original EEG signals from 4 channels within a month as input	This model could successfully promote EEG signals and classify drivers’ cognitive workload with high accuracy
Bhardwaj [164]	Cognitive	A highly accurate, EEG based driver fatigue classification system to reduce fatigue related road accidents	Based on different indicators, the accuracy of the deep learning automatic encoder was as high as 99.7%

CNN: Convolutional neural network; DL: Deep learning; MF: Multimodal fusion; MDD: Major depression disorder; RNN: Recursive neural network; AES: Dynamic electrode set.

## Data Availability

Not applicable.
